# Emotion estimation from video footage with LSTM

**DOI:** 10.3389/fnbot.2025.1678984

**Published:** 2026-02-06

**Authors:** Samer Attrah

**Affiliations:** Hogeschool van Arnhem en Nijmegen, Automotive and Engineering Academy, Arnhem, Netherlands

**Keywords:** emotion estimation, computer vision, social robotics, BlendFER-Lite, neural networks, Blendshapes

## Abstract

Emotion estimation is a field that has been studied for a long time, and several approaches using machine learning models exist. This article presents BlendFER-Lite, an LSTM model that uses Blendshapes from the MediaPipe library to analyze facial expressions detected from a live-streamed camera feed. This model is trained on the FER2013 dataset and achieves 71% accuracy and an F1-score of 62%, meeting the accuracy benchmark for the FER2013 dataset while significantly reducing computational costs compared to current methods. For the sake of reproducibility, the code repository, datasets, and models proposed in this paper, in addition to the preprint, can be found on Hugging Face at: https://huggingface.co/papers/2501.13432.

**JEL Classification:**

D8, H51

**MSC Classification:**

35A01, 65L10, 65L12, 65L20, 65L70

## 1 Introduction

In social robotics, many parts of a robot system are integrated into a single system. Typically, the more complex the system is, the better it performs tasks, which are more commonly focused on elderly care or patient care. In addition to skeletal and motion structures, a robot's subsystems include interaction modules and sensory functions, such as speech, vision, and hearing. Since human emotion is a large part of any interaction between two or more humans, it is important for robots to interpret human emotion using sensory inputs such as speech, tone of voice, facial expressions, body poses, hand gestures, gaze direction, and head orientation. In this work, we focus solely on emotion estimation from facial expressions, given their importance (Marechal et al., 2019).

Emotion estimation from facial expressions, more commonly referred to as facial emotion recognition (FER), generally involves three steps: (1) *face detection*: identify a face in a camera stream or image and localize its boundaries; (2) *feature extraction*: identify and extract the most relevant information from the previously detected face. Many approaches exist; more popular is by setting key points as a vector for the most relevant parts of the face, such as the tip of the nose and the ends of the mouth in two-dimensional or three-dimensional coordinates, and (3) *expression classification*: using extracted features as inputs for a classification model that predicts the emotion conveyed by the facial expression.

The proposed emotion estimation system functions as a feedback mechanism that helps drive a conversation or interaction between a human and a robot. The system enables a robot to detect real-time human reactions through facial expressions and potentially change the topic, approach, or action of the conversation.

The scope of this research was *limited by the availability of data and computational resources*. Consequently, emotion classification was restricted to three classes instead of the original seven proposed previously (Happy, Sad, Angry, Afraid, Surprise, Disgust, and Neutral) (Ekman and Friesen, 2003). These three classes had labels (happy, sad), while all other emotions were grouped into a general category under an undefined label (Unknown).

The system development process begins with a full data processing pipeline, which includes loading, cleaning, augmenting, extracting features, and visualizing data. The data are then input into the BlendFER-Lite model for training. During inference, the trained model is integrated into the Gaze Project^1^, where it facilitates facial detection/localization and feature extraction. Then, the trained model classifies facial expressions based on the features.

Some considerations should be noted. First, the FER2013 (Goodfellow et al., 2013) dataset is considered a challenge dataset, i.e., it was not built or optimized for research purposes. It contains a group of non-relevant images, such as animations and images with covered faces, that do not provide useful information to the model. Additionally, model training and testing are performed on a laptop equipped with an NVIDIA GeForce RTX 3050 Laptop GPU, running on the Linux Ubuntu operating system, and the programming language used is Python, under the Apache-2.0 license.

This study serves as a *proof of concept (POC)* for a research paper that will be discussed in the future work Section 8. In the context of emotion estimation for embedded systems in social robotics, the main contributions of this study are as follows:

1. Building BlendFER-Lite into a compact and cost-effective model suitable for inference on a microcomputer, while considering the spatial and temporal aspects of facial expression.
2. Using the MediaPipe library Face Landmarker task, which is for face localization and feature extraction, and incorporating Blendshapes (Lugaresi et al., 2019a) as features, is a technique rarely employed in FER applications.

The remainder of this article is organized as follows: Section 2 covers related work, Section 3 details the methods, Section 4 discusses the ablation studies, Section 5 presents the results, Section 6 discusses the results and findings of the study, Section 7 concludes the study, and Section 8 presents the future work to be based on this study.

## 2 Related work

Facial emotion recognition has been an active research area for a long time (Ekman and Friesen, 2003). The main challenges for building an emotion estimation system are Zafeiriou et al. (2017).

- The complexity of the emotion patterns.
- The emotions are time-varying.
- The process is user- and context-dependent.

Many methods exist for facial detection and tracking, such as the Haar cascade algorithm, and other methods for feature extraction, such as the classical algorithms *Histogram Oriented Gradient* (HOG) and *Scale Invariant Feature Transform* (SIFT) (Gautam and Seeja, 2023; Kumar et al., 2016), or, more recently, the *Convolutional Neural Network* (CNN) (Li and Deng, 2020) and *transformer encoders* (Min et al., 2024) have been employed to detect faces and extract features. Classification models also employ CNNs, *Recurrent Neural Networks* (RNNs), or hybrid architectures (Li and Deng, 2020; Leong et al., 2023).

### 2.1 Feature extraction

Feature extraction can be considered a special type of data dimensionality reduction aimed at identifying a subset of informative variables from image data (Egmont-Petersen et al., 2002). The most common approach for extracting high-quality features from facial images involves estimating facial landmarks (Kumar et al., 2025; Belmonte et al., 2021; Shaila et al., 2023). One approach involves identifying the nose tip and segmenting it by cropping a sphere centered on it. Then, the eyebrows, mouth corners, eye corners and possibly many other facial landmarks are identified on the segmented sphere. Finally, by measuring the distances between landmarks (An et al., 2023) and changes in their positions, facial expressions can be interpreted (Farkhod et al., 2022).

#### 2.1.1 History

Looking back on the evolution of FER, one of the methods used to extract features is the HOG (Gautam and Seeja, 2023), which determines edge presence, direction, and magnitude. Another method is SIFT (Kumar et al., 2016), which follows a four-step process: (1) constructing the scale space, (2) localizing crucial points, (3) assigning orientation, and (4) assigning unique fingerprints. A feature extraction method still widely used today is the *Support Vector Machine* (SVM) (Cortes and Vapnik, 1995; Leong et al., 2023).

More recently, feature extraction has been performed using deep neural networks (Li and Deng, 2020; Leong et al., 2023), such as *fully connected networks* (FCNs) and CNNs, which construct models consisting of multiple layers and hyperparameters. The complexity of the data, the required performance, and the available computational resources influence which model is implemented.

#### 2.1.2 MediaPipe

This study used the MediaPipe (Lugaresi et al., 2019a,b) library for facial recognition, tracking, feature extraction, and landmark detection. Using the Face Landmarker task could yield three outputs per image or frame: 468 3D landmark vectors, 52 Blendshapes, and facial transformation matrices. In previous works (Bisogni et al., 2023), two approaches were proposed for feature extraction, one of which involved using MediaPipe to extract features from images. The results indicated that using this library can be more effective than deep learning networks, such as CNNs, for subtle facial expressions. The more intense the facial expressions are, the smaller the performance gap between the two approaches. In the most extreme cases, CNNs perform better across most emotions.

**Figure 1:**
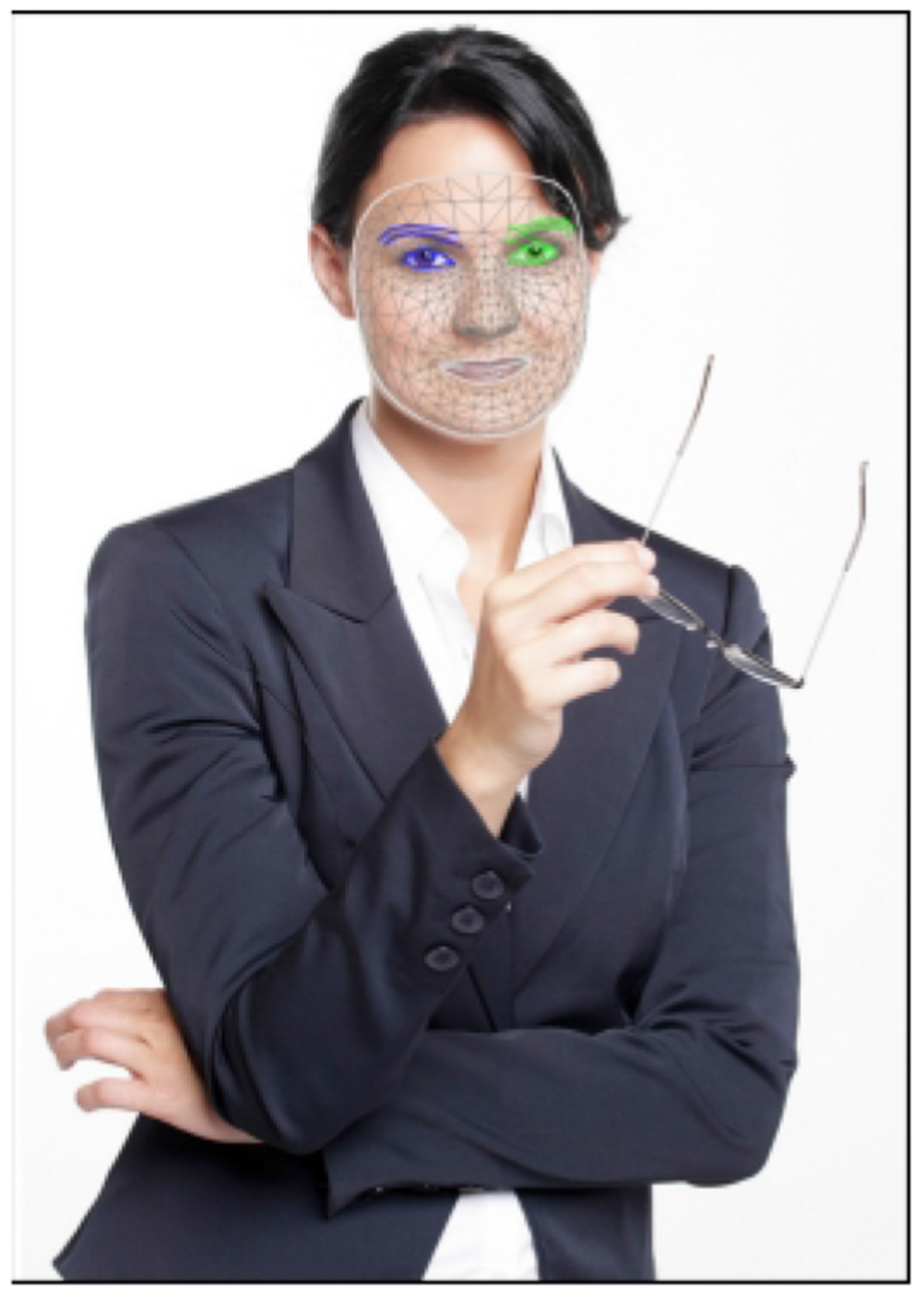
Image annotated with MediaPipe.

In another study (Savin et al., 2021), MediaPipe was compared to OpenFace for landmark detection. OpenFace (Baltrušaitis et al., 2016) uses OpenCV to detect 68 facial landmarks, whereas MediaPipe uses TensorFlow (Abadi et al., 2016) to identify 468 landmarks that are arranged in fixed quads and represented by their coordinates (x, y, z). Figure 1 shows an image annotated with the landmarks used for facial expression recognition.

Another study is Kleisner et al. (2024), which proposes adding a CNN to process MediaPipe 3D landmark vectors and improve landmark placement precision, working on 2D facial photographs.

#### 2.1.3 Blendshapes

The second output of the MediaPipe face landmark detection task model consists of Blendshapes, which provide an approximate semantic parameterization and a simple linear model for facial expressions (Lewis et al., 2014). This technique originated in the industry before gaining traction in academia and has been widely used in computer graphics. Although the Blendshapes technique is conceptually simple, developing a full Blendshapes face model is computationally intensive. To express a complete range of realistic expressions, one face might require more than 600 Blendshapes.

The construction of a single Blendshapes model was originally guided by the *Facial Action Coding System* (FACS) (Ekman and Rosenberg, 1997). This system enables manual coding of all facial displays, known as action units, and more than 7,000 combinations have been observed. The FACS action units are the smallest visibly discriminable changes in a facial display, and combinations of FACS action units can be used to describe emotional expressions (Ekman, 1993) and global distinctions between positive and negative expressions.^2^

**Figure 2:**
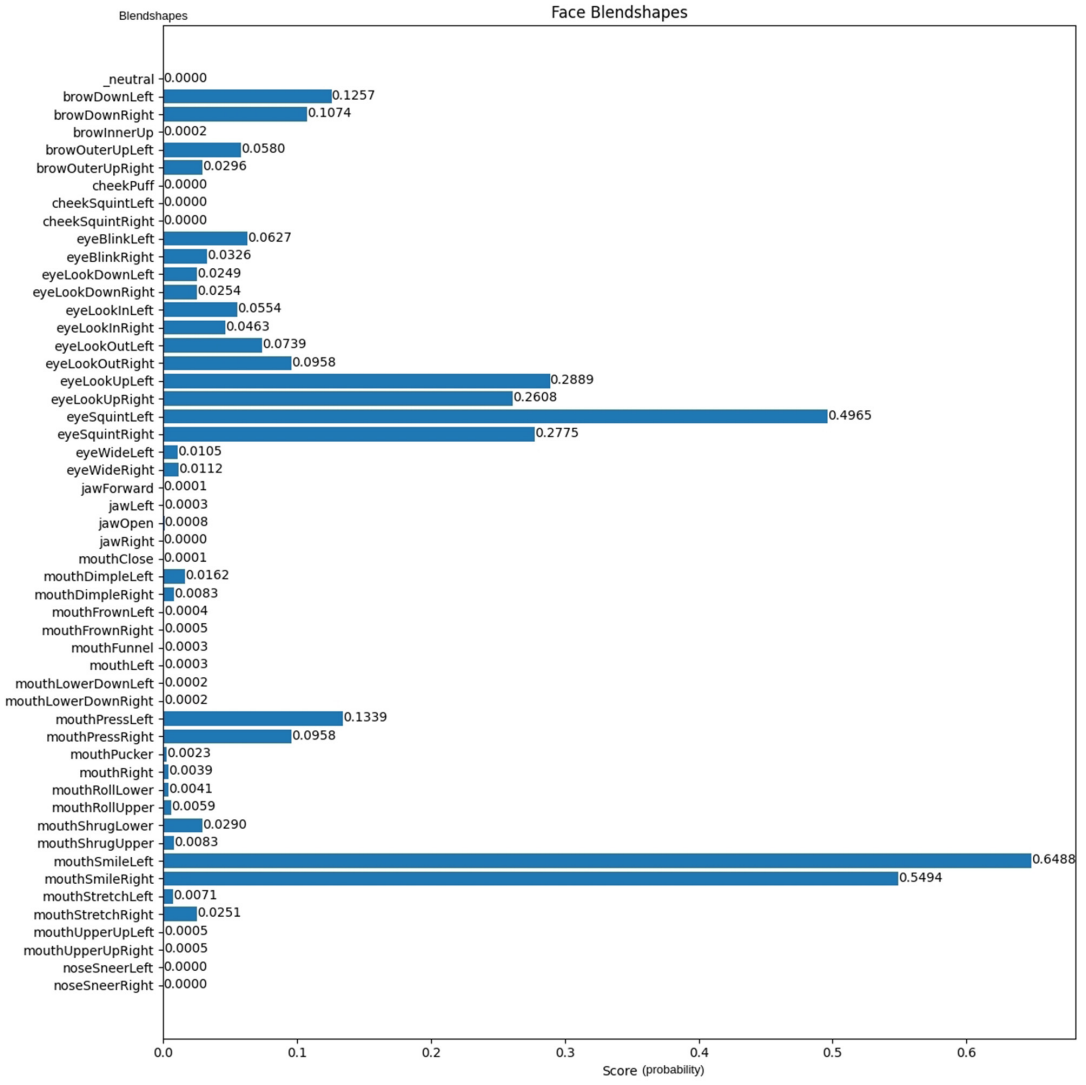
Histogram for the Blendshapes of the image in Figure 1 via MediaPipe.

MediaPipe incorporates a set similar to the ARKit face Blendshapes,^3^ which consists of 52 Blendshapes describing facial parts and expressions. These are quantified with probability scores from 0 to 1, indicating the presence of specific Blendshapes, as shown in Figure 1, while Figure 2 presents a Blendshapes histogram.

### 2.2 Emotion classification

For emotion classification, many approaches have been used in research, such as support vector machines (SVM) (Kumar et al., 2016), which have been implemented with linear as well as radial basis functions, and *stochastic gradient descent* (SGD) classifiers, which achieved approximately 95% accuracy on the *Radboud Faces Database* (RaFD) (Langner et al., 2010). Bisogni et al. (2023) presented a comparison between two approaches, namely, a MediaPipe-SVM and a CNN-LSTM. MediaPipe and the CNN were employed for feature extraction, and the SVM and LSTM (Computation, 2016) were used for classification. Experiments have shown that features extracted using MediaPipe are superior to those extracted by the CNN. Consequently, the classification performance of the first combination is better than that of the second approach.

One approach in Huang et al. (2023) used ResNet (He et al., 2015) blocks in addition to the Squeeze-and-excitation network (Hu et al., 2018) for expression classification and demonstrated the improvements that can be achieved using the correct transfer learning approach. It also used the suggested model to examine the important features and the location of the major facial information using feature maps, which will be further discussed in Section 6.

Another type of image classification method adopts CNNs. Gautam and Seeja (2023) used a sequential model of three convolutional layers and a dense layer to classify input features, achieving high accuracy on the dataset used.

Since the model being developed is designed for video processing, where data are sequential, as stated in Goodfellow (2016), choosing an appropriate RNN is crucial. Just as a CNN is a neural network specialized for processing a grid of values, such as an image, an RNN is a neural network specialized for processing a sequence of values or sequential data.

When working with sequential data, such as videos, and considering temporal dependencies between images, an RNN is the best choice (Li and Deng, 2020). The use of an LSTM, which is a special type of RNN, aims to *address the vanishing gradient and exploding gradient problems* that are common in RNN training; Jain et al. (2018) compared the results, and the network including a CNN part and an RNN part delivers better accuracy than a network with only CNN layers, which is approximately 20% higher in accuracy.

Another study discussing RNNs is Leong et al. (2023), where a CNN-LSTM architecture was proposed as a facial recognition system that can understand spatiotemporal properties in video.

## 3 Methods

This section discusses the approach to building the emotion estimation system, starting with the dataset used and the data processing techniques, then building and optimizing the model, and finally evaluating and testing the model's performance.

### 3.1 Dataset

Since the model being developed is deliberate for video-based inference, selecting a video dataset with different facial expressions was the first priority. Li and Deng (2020) found a group of datasets, some of which include videos, such as *MMI* (Pantic et al., 2005; Valstar et al., 2010) and *AFEW7.0* (Dhall et al., 2017). However, due to computational constraints and the intensive data preprocessing required for video-based datasets, we opted to use an image-based dataset instead.

Among the datasets considered, *MultiPIE* (Gross et al., 2010) was included due to its image quality and size. During the search, we also considered the Radboud Faces Database RaFD (Langner et al., 2010) for the structure of the database and the quality of the distribution. Another choice was *AffectNet* (Mollahosseini et al., 2017), owing to its suitable size and popularity as a benchmark to evaluate the performance of models in many studies and experiments.

In this study, the *FER2013* dataset (Goodfellow et al., 2013) was used, which was chosen for its simplicity. This dataset contains around 35K 48x48 grayscale images, a sufficient number that are easy to obtain from this open-access dataset.

The dataset was downloaded from Kaggle^4^ *in .csv format*. The data include the class, image pixel array grayscale values, and the data split as columns and training examples as rows.

The FER2013 dataset (Goodfellow et al., 2013) is categorized into seven classes: happy, sad, angry, afraid, surprise, disgust, and neutral. The full dataset is split into three groups: training, public testing, and private testing, which serve as the training, validation, and test sets, respectively. The distribution of images across these subsets is shown in Table 1.

**Table 1:** FER2013 dataset: classes and splits.

**Class - Split**	**Full**	**Training**	**Public test**	**Private test**
Happy	8,989	7,215	895	879
Sad	6,077	4,830	653	594
Angry	4,953	3,995	467	491
Afraid	5,121	4,097	496	528
Surprise	4,002	3,171	415	416
Disgust	547	436	56	55
Neutral	6,198	4,965	607	626

The table shows that (1) the happy, sad, and neutral classes have the highest number of samples, whereas the disgust class has the lowest representation, and (2) the *public test and private test splits are well-suited for use as the cross-validation set and test set*, respectively. In the next subsections, the data processing techniques applied to restructure the database in a form that best serves the requirements of the model being built are discussed.

### 3.2 Data processing and cleaning

Since the FER2013 dataset (Goodfellow et al., 2013) was originally designed as a challenge dataset, additional data processing steps are required to adapt it for research and real-world robotic applications. To ensure its suitability for emotion estimation in this context, a series of data cleaning and preprocessing steps must be applied.

#### 3.2.1 Creating training data classes

The first step in data processing involves splitting the dataset into three subsets: training, validation, and test. Owing to class imbalance, we decided not to use the full set of available training class images; instead, we included a portion of the available samples, as shown in Table 2.

**Table 2:** Training set classes counts.

**Class**	**Happy**	**Sad**	**Angry**	**Afraid**	**Surprise**	**Disgust**	**Neutral**
Count	4,000	4,000	1,500	1,500	1,500	Maximum possible	1,500

These values were chosen because the model focuses only on classifying happy, sad, and unknown classes, rather than the full seven emotions in the dataset. Assigning 4,000 samples to the main emotion classes *ensures a more balanced dataset*. The majority of other classes are allocated 1,500 samples each, keeping their combined total at 6,000, in addition to approximately 400 images for the disgust class. This distribution *provides a sufficient number of training examples while ensuring that the unknown class remains aligned with happy and sad emotions*.

Table 1 shows that including more training examples from each class is possible for some classes, but including all examples could lead to imbalanced data representation and a lower-quality distribution.

#### 3.2.2 Readability by MediaPipe

To clean the dataset and prevent runtime errors caused by images that MediaPipe cannot detect, a detection program was developed. All images in the training set were processed with MediaPipe, and undetectable images were excluded from the dataset.

This process resulted in the following counts for each class, as shown in Table 3.

**Table 3:** Number of images MediaPipe could not detect in the FER2013 dataset.

**Class**	**Happy**	**Sad**	**Angry**	**Afraid**	**Surprise**	**Disgust**	**Neutral**
Counts	377	835	597	616	239	72	261

**Figure 3:**
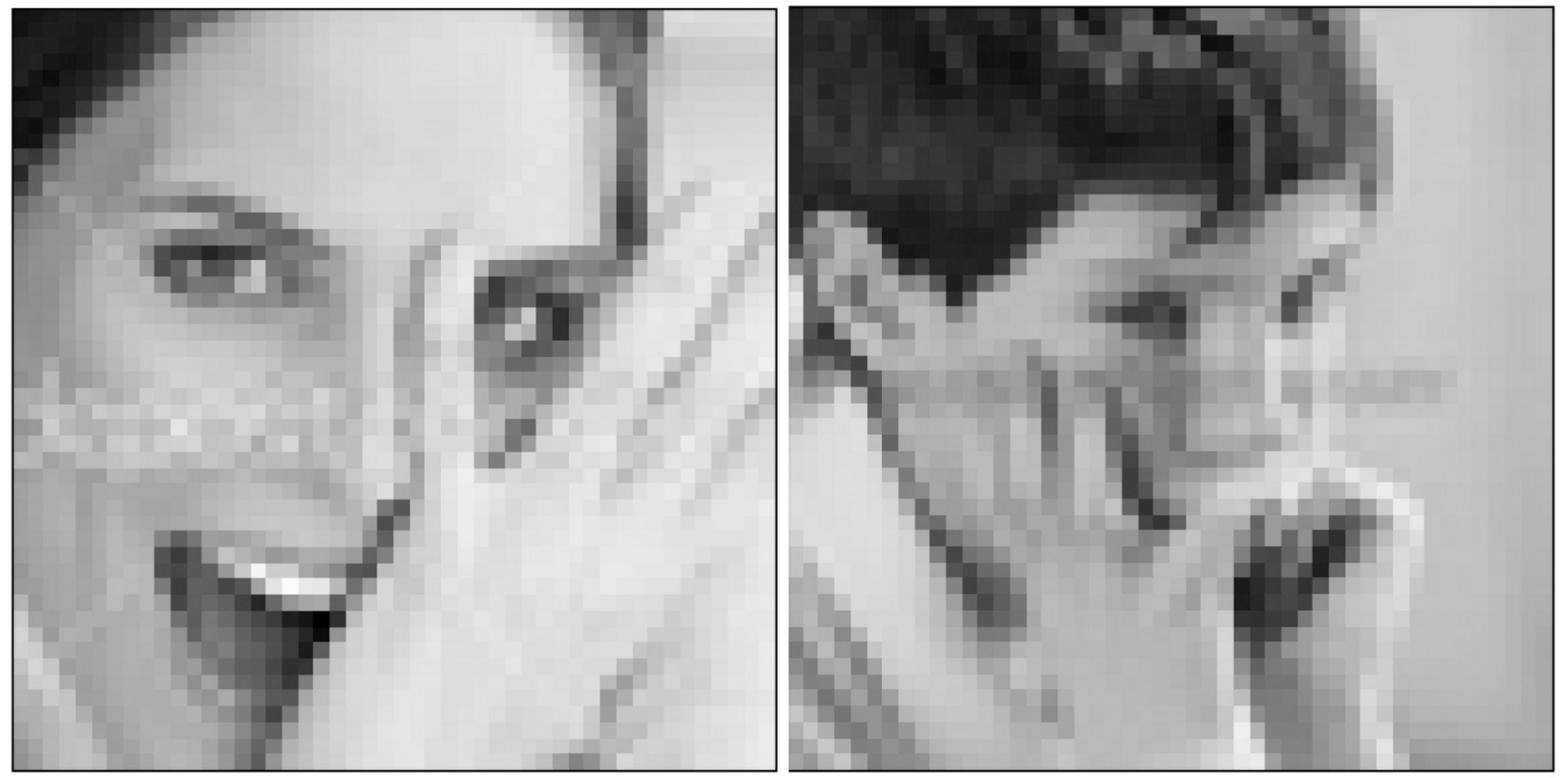
Example of unidentifiable samples from the FER2013 dataset. This figure displays representative 48 × 48 pixel images from the FER2013 dataset where faces were not successfully detected by the MediaPipe framework, primarily due to insufficient facial exposure or occlusion.

As described earlier, MediaPipe detects the main parts of the face and estimates landmark vectors and blendshapes for each image. If a part of the face is obstructed, this process becomes infeasible. Images with occlusions, such as those in Figure 3, are considered undetectable.

#### 3.2.3 Indexing the test set

This step followed a few experiments that revealed an unexplainably high error rate in model performance. *As a tracking method*, a column was added to the test set in the CSV file, which assigns a number to each image in the FER2013 dataset. This number remains associated with the image throughout data processing and is transferred to the image's Blendshapes when the Blendshapes dataset (Attrah, 2025b) is created.

This step helps track images with high error rates when the model is evaluated on the test set, facilitating visualization of the images or the creation of different types of plots to identify patterns in the images causing the errors.

#### 3.2.4 Augmenting the training set

To increase the number of training images and the variety of poses, and thereby *improve the model's generalizability*, data augmentation was applied. By generating new examples (More, 2016) with random transformations, the model's generalizability was enhanced. The augmentation techniques that were used are as follows:

1. Random horizontal slip.
2. Random rotation of 0.2 × 180 degrees in the clockwise direction and counterclockwise direction.

These techniques are based on the model's use case. Since it is designed for a social robot, horizontal flipping will enable the robot to *interpret reflections* and provide a valid facial image from a different camera angle. Additionally, random rotations improve model performance by accounting for *natural head tilts and rotations*, which are commonly observed in human interactions. For a robot, the ability to detect and classify facial expressions from various angles is crucial for real-world applications.

Processing the image dataset through augmentation yields a new, extended dataset with more than 20,000 images across the three classes described earlier.

#### 3.2.5 Blendshapes dataset

Using MediaPipe to detect faces in a video stream requires extensive data processing before the data are fed into the classification model. To integrate the model with a face detection program, the model needs to be trained to interpret the program's output, i.e., the Gaze project's Blendshapes. Consequently, the full dataset is converted into a Blendshapes dataset (Attrah, 2025b), which is used to train the model.

Using Blendshapes was preferred over the 3D landmark vectors and the base 48 × 48 images to *save a significant amount of computation* during inference. This is because using the 3D landmark vectors is equivalent to using 1,404 features to represent the image, and the features of the 48x48 images sum up to 2,304 while using the Blendshapes equals having only 52 features. Further possible processing steps that were experimented with are in the Supplementary material.

### 3.3 BlendFER-Lite architecture and training

The classification model BlendFER-Lite is primarily built using long short-term memory (LSTM) layers (Computation, 2016), with the exception of the last dense layer, which employs softmax activation to produce classification scores from the output of the final layer.

The decision to incorporate LSTM layers in building the BlendFER-Lite model was made for the following reasons:

1. The BlendFER-Lite model is designed to be integrated into a video stream and to classify faces, meaning it operates on a time-series basis, where each input depends on the sequence of preceding inputs. Given this dependency, using one type of (RNN) (Goodfellow, 2016) is intuitive, as it retains information from past states in addition to the current input.
2. Another reason for this choice is that LSTM is a type of gated recurrent unit (GRU) (Chung et al., 2014). One of its advantages is an internal gating mechanism that helps filter out irrelevant features, allowing the model to focus on the most critical ones.
3. LSTM stands for long short-term memory. A key advantage of this network over other types of recurrent neural networks is its ability to maintain long-term dependencies effectively, not just short-term ones, as is the case with RNN (Medsker et al., 2001) and GRU networks.

Once the LSTM layer was selected, the next step was designing the model architecture. Several key factors were considered, such as accuracy, recall, precision, latency, and model size. After a few experiments to determine the best parameters, the **Keras Tuner** (O'Malley et al., 2019) was employed as an architecture search framework to identify the best values for the hyperparameters. The search space was defined based on prior experiments and empirical intuition^5^.

**Figure 4:**
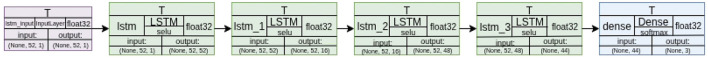
BlendFER-Lite model structure.

The final model was trained for 5,000 *epochs*, which took approximately 2 days. The *batch size* was 128, and the architecture was plotted using a design similar to the Keras utils model plotting function, as shown in Figure 4. *The optimizer* used for training was AdamW (Kingma, 2014; Loshchilov, 2017), with a *learning rate* of 1.09e-06, a *global clipping norm* of 1, and *AMSGrad* (Reddi et al., 2019). The adopted callbacks were the *checkpoint callback* and *early stopping callback*.

For the loss function, *categorical cross-entropy* was used to encode the labels of the images as one-hot vectors, and the metrics for evaluation were loss, categorical cross-entropy, categorical accuracy, and the F1-score. Other details and experiments are available in the Supplementary material.

### 3.4 Model evaluation

The model evaluation process is conducted for every set of weights produced by the checkpoint callback and shows improved training and validation performance metrics. Approximately every 100 epochs, a model's weights are saved.

Each model is evaluated by predicting the Blendshape labels for test dataset images and comparing them to the ground truth labels. A loss, categorical cross-entropy, categorical accuracy, and F1-score are calculated for the model, and the F1-score is used as the main metric for optimization.

## 4 Ablation studies

To better understand the BlendFER-Lite model and its potential applications, several ablation studies were conducted as part of the experiments. These studies aimed to determine the optimal number of Blendshapes, the most effective model type, and the best loss function.

### 4.1 Selection of relevant Blendshapes

To improve the efficiency of the model, reduce computational requirements, and output quality classification results with *less processing time and memory usage*, a set of Blendshapes returned from MediaPipe is disregarded. Since the model processes detected faces in each video frame, a total of 52 Blendshapes, similar to the ARKit face Blendshapes, are processed.

**Figure 5:**
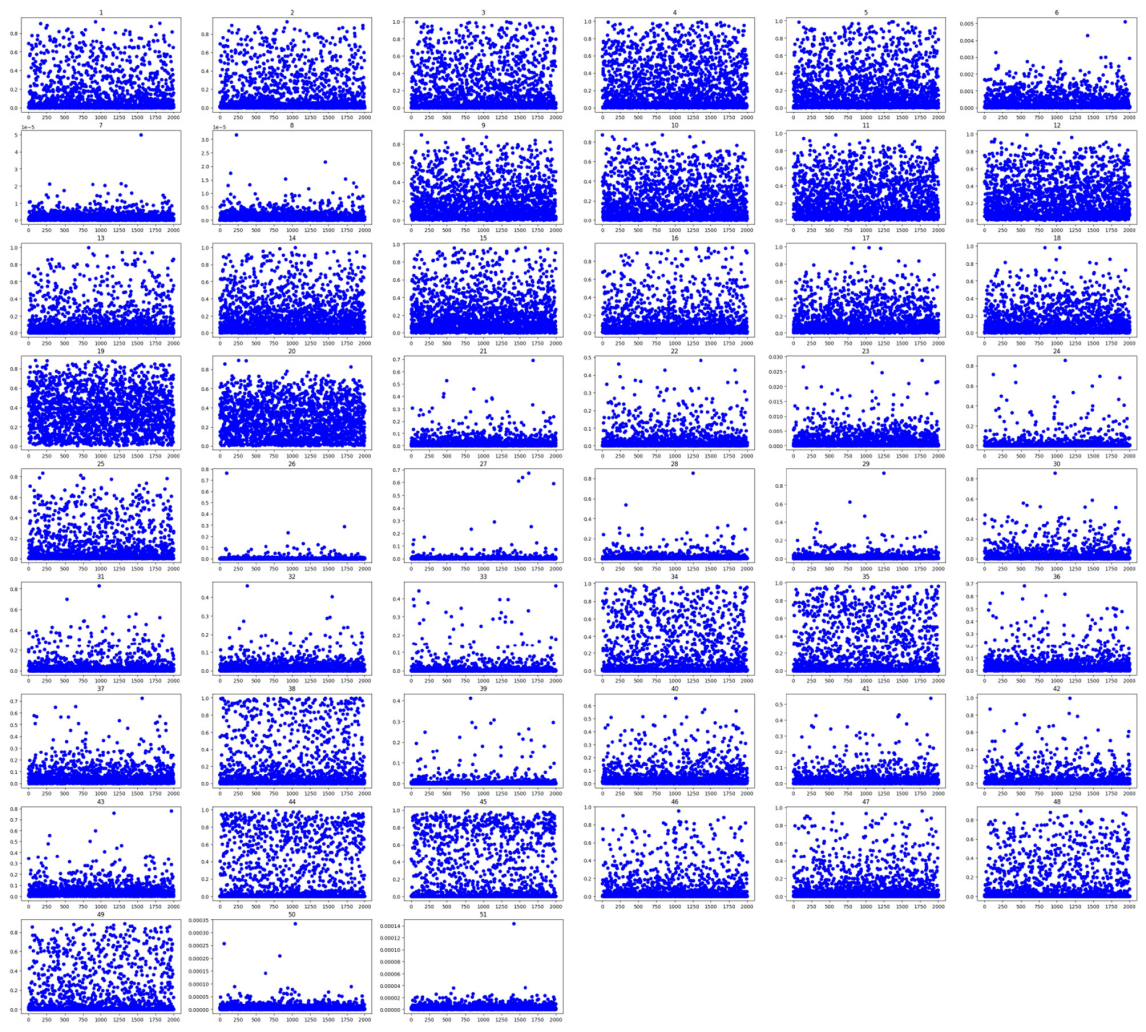
Blendshapes scatter plot for all images of the training dataset.

This approach was developed as a result of observing, as might be obvious in Figure 2, that some Blendshapes do not have a score. Testing the whole dataset (Attrah, 2025b) revealed that some of the Blendshapes do not have scores across all images, as can be found in Figure 5, where each subplot rectangle corresponds to a Blendshape and the blue dots are for the images. It is clear that in some subplots, the dots are spread across the entire rectangle almost uniformly, which indicates that it has coverage of the range 0–1, which are the values of the Blendshapes while in other subplots, the blue dots are concentrated in the lower half of the rectangle.

The research considered two strategies: (1) count the number of times a certain Blendshape has a zero score and discard it if it has a high count and (2) set a threshold value and count how many times each of the Blendshapes has a score that exceeds this threshold.

After testing various values for the high-count threshold in the first approach and different threshold values in the second approach, 0.4 was selected as the cutoff threshold, and 100 was set as a high-count number. Thus, for any Blendshape, if its score does not exceed 0.4 in more than 100 images in the dataset (Attrah, 2025b), it is discarded.

This choice was made for the following reasons:

- Counting zero values and making decisions on the basis of these criteria would have resulted in a small and non-relevant number of Blendshapes being discarded, which would not have improved the efficiency of the model.
- Using the second approach and setting a threshold higher than 0.4 would have resulted in discarding Blendshapes with a wide range of probabilities. These Blendshapes contribute valuable information to the model during training and removing them would result in a more compact model but reduced accuracy and performance.
- Setting a count higher than 100 would have resulted in a significant decrease in the number of Blendshapes being used, which would make it more difficult for the model to learn.

These reasons were validated through experimentation: training models on different data collections, testing their performance and size, and then making decisions for each number mentioned.

This process used 27 of the 52 Blendshapes listed as important in the Supplementary material, reducing the model size while keeping performance metrics, such as accuracy and F1-score, unchanged.

For further clarification, Figure 5 shows the scatter plot for the 52 Blendshapes. When discussing the importance of the Blendshapes table in the Supplementary material, highlight how many of its entries exhibit low values. This finding suggests that certain Blendshapes contribute minimal information and can be omitted.

### 4.2 Dense neural network model

To construct a faster model with shorter prediction time (latency) than the LSTM network, another, simpler network was built, with a fully connected (dense) layer as the only layer in the model. After a few optimization experiments, the model achieved high performance. However, when integrated into a real-time system using a camera stream, it lacked acceptable stability and oscillated between the classes without any change in the camera view, resulting in predictions with insufficient accuracy. This is because the layer does not consider the time dimension in the input data, which is well handled by the LSTM layer, as discussed further in the Supplementary material.

### 4.3 CCE and MSE loss functions

In earlier stages of the research, the mean squared error (Error, 2008) loss function was used to train the LSTM model. However, because the MSE function measures the distance between the prediction and the ground truth rather than the probability difference, it is better suited for regression applications. Therefore, the loss function had to be changed after a certain point.


$$\begin{array}{l} MSE=\frac{1}{n}\overset{n}{\underset{i=1}{\sum }}{\left(y_{i}-{\hat{y}}_{i}\right)}^{2} \end{array}$$ (1)

Unlike the cross-entropy, categorical cross-entropy measures the difference between the predicted probability of the correct category and the ground-truth label, making it more suitable for classification tasks. Given that the application involves three classes, categorical cross-entropy was chosen.


$$\begin{array}{l} CCE=-\overset{N}{\underset{i=1}{\sum }}y_{i}\log\left({\hat{y}}_{i}\right) \end{array}$$ (2)

To address class imbalance in the dataset, a potential improvement was using categorical focal cross-entropy (Lin, 2017). Although it was originally designed for object detection, previous research has shown that categorical focal cross-entropy improves classification model performance.


$$\begin{array}{l} FL=-\alpha {\left(1-\hat{y}\right)}^{\gamma }CCE\left(y,\hat{y}\right) \end{array}$$ (3)

## 5 Results

The results obtained from the BlendFER-Lite model were in the top five in the classification benchmark of the dataset of the Papers With Code website^6^ taking into consideration the requirement of the model to have small size, and to have a more accurate comparison, the BlendFER-Lite model is compared to models with no extra training data and a condition of being a non-transformer neural network only. Such as the models mentioned in Table 4.

**Table 4:** Benchmark models.

**Model name**	**Accuracy**
LocalLearning BOW (Goodfellow et al., 2013)	67.48%
DeepEmotion (Minaee et al., 2021)	70.02%
BlendFER-Lite (Proposed model)	**71.99%**
Ad-Corre (Fard and Mahoor, 2022)	72.03%
VGGNet (Khaireddin and Chen, 2021)	73.28%
ResNet18 with tricks (Yuan, 2021)	73.70%

For more information, check the Papers with Code link mentioned on the previous page.

Where the Table 4 shows that the BlendFER-Lite model is showing enhanced performance by around 2% compared to the LocalLearning BOW and the DeepEmotion models, and given the minimal architecture and model size, the enhancement is greatly considerable. While the Ad-Corre, VGGNet, and ResNet18 with tricks deliver higher accuracy, they do not surpass a 2% cap in improvement, in addition to the depth of the model making them resource-consuming. The best result metrics, aside from accuracy, obtained from the model on the test set which consists of *Happy: 291, Sad: 245, and Unknown: 1110 images' blendshapes* are as follows:

- Loss = 0.6238.
- Categorical cross-entropy = 0.6235.
- Categorical accuracy = 0.7199.
- F1-score = 0.6298.

For a more detailed analysis, the model's confusion matrix is shown in Table 5.

**Table 5:** Confusion matrix.

	**Predicted class**			
	**Class**	**0**	**1**	**2**
True class	0	251	35	5
	1	110	850	150
	2	21	140	84

Shows the classes as 0: happy, 1: unknown, and 2: sad.

The confusion matrix indicated that the happiness emotion achieved the highest classification accuracy, with 251 correctly classified instances out of 291 (a miss rate of 40). In contrast, the unknown class exhibited a correct classification rate of 850 out of 1,110 instances. Although this represents *the largest absolute number of misclassifications* (260) across the three classes, it is likely attributable to the inherent feature diversity and variability encompassed within this category. The sad emotion class demonstrated the lowest performance, with only 84 out of 246 instances correctly classified, resulting in 162 misclassified samples. While the unknown class showed the highest absolute number of misses, the sad class registered *the highest proportional misclassification rate*. It is also important to note that the test set for the sad class was the smallest of the three. Diving deep into the confusion matrix (Crall, 2023) and breaking down each part of it shows the happy, unknown, and sad classes as in Table 6.

**Table 6:** The counts of the types of classifications extracted from the confusion matrix.

**Class**	**0**	**1**	**2**
True Positives (TP)	251	850	84
True Negatives (TN)	934	335	1101
False Positives (FP)	40	260	161
False Negatives (FN)	131	175	155

From these numbers, we can see that the unknown class has the highest counts in all parts, indicating consistent performance across all classes and inaccuracy corresponding to the total count of the class sample. Additionally, finding the precision and recall can be easily done using the Equations 4, 5


$$\begin{array}{l} Precision=\frac{TP}{TP+FP} \end{array}$$ (4)

$$\begin{array}{l} Recall=\frac{TP}{TP+FN} \end{array}$$ (5)

$$\begin{array}{l} F1=2*\frac{Precision*Recall}{Precision+Recall} \end{array}$$ (6)

and by calculating these metrics for each class, can yield the per-class precision and recall, as in Table 7.

**Table 7:** Precision, recall and F1-score.

**Class**	**0**	**1**	**2**
Precision	0.86	0.76	0.34
Recall	0.65	0.82	0.35
F1-score	0.74	0.79	0.34

Shows the classes as 0: happy, 1: unknown, and 2: sad.

The findings in Table 7 show that the unknown class is the easiest to detect, followed by the happy class, with the sad emotion last and the least precision, recall, and F1-score, making it difficult to recognize. which is an expected proportion resulting from the large imbalance in the dataset.

Experimenting with different architectures, hyperparameters, and longer training times did not yield improved results. However, it affected the classification accuracy metric once and the time-related stability another time. Nevertheless, the confusion matrix continued to fluctuate, showing inconsistent improvements in classification for the happy and sad classes.

At one point, the classification of the sad emotion improved, resulting in higher precision and recall, while at another, the happy class was favored, and the experiment reported in Table 5 revealed a bias toward the happy class.

To assign weights to each class, the model focused on the happy and sad classes, assigning them greater importance while allocating less weight to the unknown class, which is easily classified in all the experiments. However, some limitations prevent the use of class weights in experiments.

When the model was integrated into the test camera stream, as demonstrated in the demo video,^7^ the model outputs classifications for happy and sad. However, when facial features change, the unknown class is frequently assigned. For all other facial expressions, the unknown class remains the predicted output.

Regarding the *latency of the model*, it was measured separately from the Blendshapes extraction pipeline of mediapipe, and it shows a *mean latency of 33.47 ms* when calculated across the full test set of the FER2013 blendshapes dataset.

While the model size as a zipped .keras file is 775.385 KB (Attrah, 2025a), which is significantly smaller than the other models compared on the benchmark, and the *count of parameters of the model is 44,631 parameters*, it makes it the fastest running with the smallest memory usage compared to the best in Table 4, which is a model based on the ResNet-18 network that has more than 11M parameters.

## 6 Discussion

Considering the use case for which the model is developed, it is necessary to optimize for latency and model size, in addition to compiler metrics such as loss, cross-entropy, accuracy, and F1 score. These metrics are critical during the implementation phase when deploying the model on an edge device for real-time inference. One method experimented with involved quantizing the Blendshapes and using only 4 floating-point numbers out of 16. This significantly increased the model's speed but negatively impacted performance metrics and reduced accuracy.

Regarding the model size, models in Goodfellow et al. (2013), Min et al. (2024), An et al. (2023), Huang et al. (2023), Zhou et al. (2023), Fard and Mahoor (2022), Vulpe-Grigorasi and Grigore (2021), Yuan (2021), Khaireddin and Chen (2021), and Minaee et al. (2021) are of large size, which makes them unfit for implementation on an edge device, yet some of them might show better performance metrics. While in Febrian et al. (2023), a model of CNN-BiLSTM is presented, which makes the LSTM part of it similar to ours in processing the temporal dimension of the data but without using bidirectional layers, and that is because part of the experiments in Section 3.3 showed that it does not improve the results, only increases the time required for training. While the first part, which is a CNN, is much different from MediaPipe, although both of them are concerned with the spatial dimension of the data, and from Section 2.1.2 in Bisogni et al. (2023), MediaPipe shows superior results to a CNN model for feature extraction.

Section 4.1 suggested and validated an approach to decrease the number of Blendshapes included in representing the face and used a statistical approach to choose the best Blendshapes to use, and it showed the Blendshapes to be included are a *majority from the brows, eyes, and mouth*, and as shown in the Supplementary material, while in Huang et al. (2023) generates feature maps that show that *the mouth and the nose* are the parts with the most information and importance for classification, and this difference might be resulting from the difference in the datasets and the approach used, since Blendshapes are a different concept from the feature maps suggested. Also, notice that only two Blendshapes of the 52 in the MediaPipe blendshapes set represent the nose, which might be hiding some information.

## 7 Conclusion

This article presents the building of BlendFER-Lite, a model to be implemented on a microcomputer in a social robot to detect faces in a camera video stream, extract Blendshapes as features from the face using MediaPipe, and classify facial expressions based on the emotions they represent, whether they are happiness, sadness, or unknown.

Demonstrated that a 4-layer LSTM model is a suitable architecture to classify the Blendshapes of faces in camera stream frames. The model showed good temporal stability. Note that good results were obtained from the video stream even after the model was trained on an image dataset. In addition, the model achieves results competitive with the dataset's benchmark, with no loss in accuracy throughout the feature extraction and classification pipeline. This approach saves considerable memory and computation in comparison to other available methods.

Besides that, conducted ablation studies and suggested methods to further improve the model's resource efficiency and performance.

## 8 Future research

Based on this research, a new model will be built and trained mainly on the Aff-Wild2 Dataset (Kollias et al., 2019b; Kollias and Zafeiriou, 2019; Kollias et al., 2019a, 2020; Kollias and Zafeiriou, 2021a,b; Kollias et al., 2021; Kollias, 2022, 2023; Kollias et al., 2023; Zafeiriou et al., 2017) and tested on other datasets in addition to Aff-Wild2, to deliver results on facial expression classification, action units, and valence/arousal. and will use techniques discussed and implemented in this research paper, as well as more advanced architectures and models, such as transformer-based models (LLMs, VLMs, and MLLMs). Afterward, an expansion to work on another research project that includes other modalities, such as voice tone, body pose, and natural language, to estimate emotions. and using compound facial expressions (Du et al., 2014) to have a more accurate and detailed estimation for human emotion.

Further studies about reducing the feature count will be conducted on other types of data and features.

## Data Availability

The datasets used for this study can be found at: Kaggle/FER2013 blendshapes dataset example (Partial) [link] and Kaggle/Challenges in Representation Learning: Facial Expression Recognition Challenge [link].

## Footnotes

1
https://gitlab.com/Hoog-V/gaze/-/tree/main/gaze_estimator_python_rpi?ref_type=heads

2 Blendshapes a facial expressions representation blog.

3
https://arkit-face-blendshapes.com/

4 Link to FER2013 Kaggle challenge data.

5
https://medium.com/p/1833f774051f

6
https://paperswithcode.com/sota/facial-expression-recognition-on-fer2013

7
https://youtu.be/RdcjA9ScBmI
