# Supplementary material for: Emotion estimation from video footage with LSTM
Source: Front Neurorobot. 2026 Feb 6;19:1678984. doi: 10.3389/fnbot.2025.1678984 (PMC12920442; doi:10.3389/fnbot.2025.1678984)
Supplement: Supplementary file 1 [file Data_Sheet_1.pdf]

Emotion estimation from video footage with LSTM Supplementary Material

1 ARKIT BLENDSHAPES

The MediaPipe blendshapes are a set of 52 blendshapes focused on the eyes and the mouth and built specifically for computer vision and edge devices development. Following is a list of the MediaPipe blendshapes in their standard order with detailed description, the descriptions are originally found at the ARKit face Blendshapes [1], besides an indicator whether the blendshape is above the 0.4 threshold set in the ablation study in subsection 4.1.

Index	Name	Description	Importance according to ablation studies
0	Neutral	Indicating whether the face is lacking expressions	No
1	Brow down left	Downward movement of the outer portion of the left eyebrow	Yes
2	Brow down right	Downward movement of the outer portion of the right eyebrow	Yes
3	Brow inner up	Upward movement of the inner portion of both eyebrows	Yes
4	Brow outer up left	Upward movement of the outer portion of the left eyebrow	Yes
5	Brow outer up right	Upward movement of the outer portion of the right eyebrow	Yes
6	Cheek puff	Outward movement of both cheeks	No
7	Cheek squint left	Upward movement of the cheek around and below the left eye	No
8	Cheek squint right	Upward movement of the cheek around and below the right eye	No
9	Eye blink left	Closure of the eyelids over the left eye	Yes
10	Eye blink right	Closure of the eyelids over the right eye	Yes
11	Eye look down left	Movement of the left eyelids consistent with a downward gaze	Yes
12	Eye look down right	Movement of the right eyelids consistent with a downward gaze	Yes
13	Eye look in left	Movement of the left eyelids consistent with a rightward gaze	Yes
14	Eye look in right	Movement of the right eyelids consistent with a leftward gaze	Yes
15	Eye look out left	Movement of the left eyelids consistent with a leftward gaze	Yes
16	Eye look out right	Movement of the right eyelids consistent with a rightward gaze	Yes
17	Eye look up left	Movement of the left eyelids consistent with an upward gaze	Yes
18	Eye look up right	Movement of the right eyelids consistent with an upward gaze	Yes
19	Eye squint left	Contraction of the face around the left eye	Yes

Table S1. The first part of the important Blendshapes for FER2013 dataset according to the criteria in the first ablation study in subsection 4.1

[1] https://developer.apple.com/documentation/arkit/arfaceanchor/blendshapelocation

20	Eye squint right	Contraction of the face around the right eye	Yes
21	Eye wide left	Widening of the eyelids around the left eye	No
22	Eye wide right	Widening of the eyelids around the right eye	No
23	Jaw forward	Forward movement of the lower jaw	No
24	Jaw left	Leftward movement of the lower jaw	No
25	Jaw open	Opening of the lower jaw	Yes
26	Jaw right	Rightward movement of the lower jaw	No
27	Mouth close	Closure of the lips independent of jaw position	No
28	Mouth dimple left	Backward movement of the left corner of the mouth	No
29	Mouth dimple right	Backward movement of the right corner of the mouth	No
30	Mouth frown left	Downward movement of the left corner of the mouth	No
31	Mouth frown right	Downward movement of the right corner of the mouth	No
32	mouth funnel	Contraction of both lips into an open shape	No
33	Mouth right	Rightward movement of both lips together	No
34	Mouth lower down left	Downward movement of the lower lip on the left side	Yes
35	Mouth lower down right	Downward movement of the lower lip on the right side	Yes
36	Mouth press left	Upward compression of the lower lip on the left side	No
37	Mouth press right	Upward compression of the lower lip on the right side	No
38	Mouth pucker	Contraction and compression of both closed lips	Yes
39	Mouth left	leftward movement of both lips together	No
40	Mouth roll lower	Movement of the lower lip toward the inside of the mouth	No
41	Mouth roll upper	Movement of the upper lip toward the inside of the mouth	No
42	Mouth shrug lower	Outward movement of the lower lip	No
43	Mouth shrug upper	Outward movement of the upper lip	No
44	Mouth smile left	Upward movement of the left corner of the mouth	Yes
45	Mouth smile right	Upward movement of the right corner of the mouth	Yes
46	Mouth stretch left	Leftward movement of the left corner of the mouth	Yes
47	Mouth stretch right	Rightward movement of the left corner of the mouth	Yes
48	Mouth upper up left	Upward movement of the upper lip on the left side	Yes
49	Mouth upper up right	Upward movement of the upper lip on the right side	Yes
50	Nose sneer left	Raising of the left side of the nose around the nostril.	No
51	Nose sneer right	Raising of the right side of the nose around the nostril	No

Table S2. The second part of the important Blendshapes for FER2013 dataset according to the criteria in the first ablation study in subsection 4.1

2 DATA PROCESSING AND ANALYSIS

Further data processing steps that were implemented and tested on the blendshapes dataset before training with the latest version are:

- Normalization for the blendshapes was considered but not used, because the empirical experiments showed that training the LSTM Computation (2016) layers without normalizing gives higher performance.

- Simplifying the Unknown class to a single emotion to reduce the number of features and patterns required from the model to learn, but that came with different challenges and excluded the majority of the dataset.

3 BLENDFER-LITE MODEL ARCHITECTURE AND EXPERIMENTS

Starting to build the BlendFER-Lite model and experimenting was to find the range that will potentially include the best hyperparameters' values for the model.

Some of these experiments were conducted on smaller models consists of two LSTM layers and using three classes namely the happy, sad, and neutral, but even with less feature density the model overfits to the training data. and from examining the coonfusion matrix can understand that the model is less able to detect the happy expressions features and more capable of detecting the sad faces. Another failure cause for the two-layer model is classifying the neutral face as sad when ever there is no eye contact for the human with the camera.

To address the overfitting behavior of the model, used kernel regularization which showed an improvement in the performance and resulted in an accuracy of 73%, but including the full dataset and renaming the neutral into unknown was more relevant to have stability and cover the full range of facial expression, instead of having the sad emotion as a classification for anger and neutral emotion as a prediction for surprise.

Another experiment, was truncating the blendshapes floating numbers to have 4 floats instead of the full range of 17, to reduce the memory utilized by the algorithm at running time, but this deletion for the floating points reduced the accuracy of the model severely, and since the dataset and model is not large, so did not experiment with other values of floats between 4 and 17.

To cover a wider range of hyperparameters values and find the best model possible, the included hyperparameters that were tested for optimization are described in the following subsections, and the code for them can be found in the history of the repository on GitHub.

3.1 Learning rate

For the Keras Tuner to run the experiments, a range for the values of the hyperparameters included in the optimization need to be set, and the range for the learning rate was 1e-6 and 1e-3, and the optimum value for the learning rate is as stated in the section 3.3 of the main article is 1.09e-06.

3.2 Activation functions

Here we recognize three types of activation function that were optimized in the model architecture.

3.2.1 The activation function of the LSTM

which is Tanh by default and has two instances in each layer was changed several times, and was included in the Keras tuner O'Malley et al. (2019) search space. The functions experimented and tested:

- ReLU
- SeLU
- Tanh
- Leaky ReLU

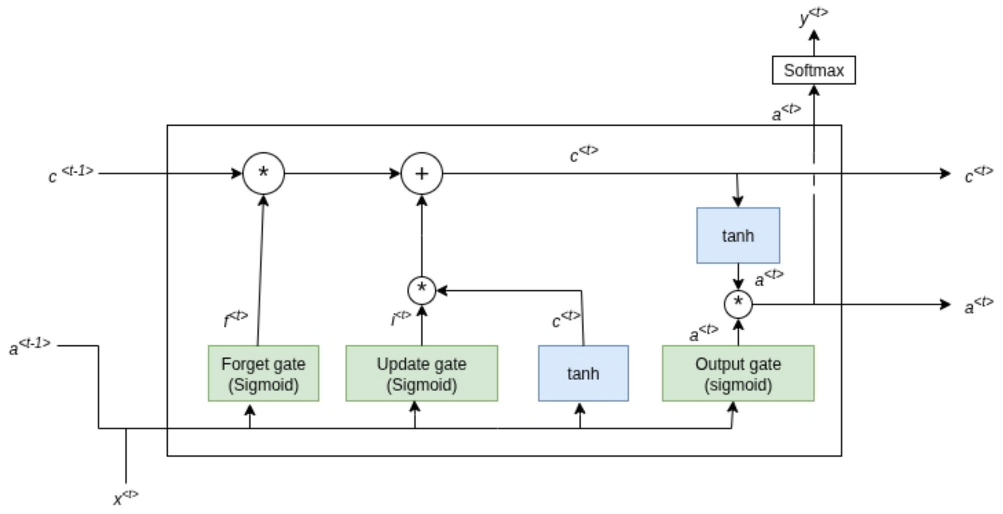

Figure S1. LSTM architecture, The annotations are: x: input features, a: activation features, c: memory state, f: forget gate state, i: update gate state, y: output

and the search space of the number of units was in the range 10 -32 with a step size of 4 while searching, the final result reached was SeLU and the insight shared in section 3.3 in the main text footnote.

3.2.2 The gate Activation of the LSTM

The LSTM layer has three gates,

- Forget gate, for managing erasing the memory of the layer, and replace it with the data.
- Update gate, to manage the layer memory (cell state) updating, and including information from the new input.
- Output gate, manages the hypothesis (hidden) output of the layer and whether to include the new time step information or output the memory only.

the default activation used for these gates is Sigmoid function, but through the experiments tested the use of other such as: Hard Sigmoid, and Softmax, but the best performance was found using sigmoid.

3.2.3 The activation function of the fully connected (Dense) classification layer

The last layer of the model is a fully connected layer to use the final output of the LSTM to generate a category label for the input, and the activation of this layer was tested with Softmax mainly, since it is a generalization for sigmoid as a multi-class function instead of a binary classification.

3.3 Kernel initializers

Many experiments were done to test the possibility of finding a kernel initializer that better fit the data distribution and a range of algorithms were tested including:

- He He et al. (2015)
- Glorot Glorot and Bengio (2010)
- Zero
- Random

and checking on the normal and uniform distributions of them for the He and Glorot, but as a result the Glorot uniform which is the default of the LSTM was used and found to deliver the best performance.

3.4 Optimizers

In the beginning of the experimentation with the model and specifically when the MSE loss function was being used the SGD optimizer was the one implemented, but when it showed limited improvement in the results, Adam was the second choice sand that is because it is most popular and efficient on many tasks.

After a few iterations and experiments, the class imbalance in the dataset was the main issue to be addressed, using AdamW was the best choice to be able to have all the available hyperparameters and experiment with changing the weight decay within the search space in the range 1e-10 to 0.0009 in addition to other parameters of the optimizers.

One of the main problems that took a long time until resolved was the exploding gradient, and using the AMSGrad and the ClipNorm was the main contributor to the solution.

3.5 Bi-directional LSTM

Part of the experiments was testing the use of Bi-directional LSTM, and that did not get improved results, but it was the opposite, since it decreased the accuracy of the model when evaluated empirically but we do not have the theoretical proof for it.

3.6 Regularization

To improve the generalizability of the model and avoid overfitting to the training data many regularization methods were used and included in the experiments:

- L1-regularization.
- L2-regularization.
- Dropout (Recurrent)
- LSTM regularizer (Kernel, Recurrent, Bias).

For the end product used the L2 regularizer as a kernel regularizer, with the regularization factor search in the range 1e-10 to 1e-5 and optimal value at 0.00000195 and that delivered the best result.

REFERENCES

Computation, N. (2016). Long short-term memory. *Neural Comput* 9, 1735–1780

Glorot, X. and Bengio, Y. (2010). Understanding the difficulty of training deep feedforward neural networks. In *Proceedings of the Thirteenth International Conference on Artificial Intelligence and Statistics*, eds. Y. W. Teh and M. Titterington (Chia Laguna Resort, Sardinia, Italy: PMLR), vol. 9 of *Proceedings of Machine Learning Research*, 249–256

[Dataset] He, K., Zhang, X., Ren, S., and Sun, J. (2015). Delving deep into rectifiers: Surpassing human-level performance on imagenet classification

[Dataset] O'Malley, T., Bursztein, E., Long, J., Chollet, F., Jin, H., Invernizzi, L., et al. (2019). Kerastuner. `https://github.com/keras-team/keras-tuner`
